# Epigenetic regulation of gene responsiveness in *Arabidopsis*

**DOI:** 10.3389/fpls.2013.00548

**Published:** 2014-01-07

**Authors:** Taiko K. To, Jong Myong Kim

**Affiliations:** ^1^Department of Integrated Genetics, National Institute of GeneticsShizuoka, Japan; ^2^Plant Genomic Network Research Team, RIKEN Center for Sustainable Resource ScienceKanagawa, Japan

**Keywords:** epigenetics, gene responsiveness, histone modification, histone variant, memory, Arabidopsis, H2A.Z, H3K4me3

## Abstract

The regulation of chromatin structure is inevitable for proper transcriptional response in eukaryotes. Recent reports in *Arabidopsis* have suggested that gene responsiveness is modulated by particular chromatin status. One such feature is H2A.Z, a histone variant conserved among eukaryotes. In *Arabidopsis*, H2A.Z is enriched within gene bodies of transcriptionally variable genes, which is in contrast to genic DNA methylation found within constitutive genes. In the absence of H2A.Z, the genes normally harboring H2A.Z within gene bodies are transcriptionally misregulated, while DNA methylation is unaffected. Therefore, H2A.Z may promote variability of gene expression without affecting genic DNA methylation. Another epigenetic information that could be important for gene responsiveness is trimethylation of histone H3 lysine 4 (H3K4me3). The level of H3K4me3 increases when stress responsive genes are transcriptionally activated, and it decreases after recovery from the stress. Even after the recovery, however, H3K4me3 is kept at some atypical levels, suggesting possible role of H3K4me3 for a stress memory. In this review, we summarize and discuss the growing evidences connecting chromatin features and gene responsiveness.

## Introduction

Gene expression patterns can change in response to a variety of endogenous and exogenous stimuli such as environmental stresses. Regulating gene responsiveness is important to alter transcription statuses when signals are present whereas not to respond when signals are absent. Recent studies in yeast, animals, and plants have suggested that transcription responsiveness can be modulated by chromatin features, such as incorporation of histone variant H2A.Z and methylation of histone H3K4. In yeast and animals, both of these marks are often found around transcriptional start sites (TSSs), which would affect transcription initiation (Raisner and Madhani, 2006; Vermeulen and Timmers, 2010). Interestingly, in a model plant *Arabidopsis thaliana*, although both of these marks are predominantly found near TSSs, they are also found throughout the transcribed regions of the responsive genes. Therefore, these chromatin features in gene bodies may play a role. Here, we will overview the importance of epigenetic regulation in gene responsiveness, focusing especially on the recent findings on *Arabidopsis* for the marks in gene bodies.

## Histone variant H2A.Z in animals and yeast

H2A.Z is conserved among eukaryotes and it is known to function in diverse genomic processes such as transcriptional regulation, telomeric silencing, DNA repair, and genome stability (Raisner and Madhani, 2006; Deal and Henikoff, 2011). H2A.Z has high frequencies of amino acid substitutions compared with canonical H2A, especially at the C-terminus domain. Although the overall structure of nucleosome core particle containing H2A.Z is very similar with that of H2A, the interaction between the H2A.Z/H2B dimer with the H3/H4 tetramer is less stable and it has abnormal surface holding a metal ion, suggesting specific roles of H2A.Z (Suto et al., 2000). H2A.Z dynamics are controlled by ATP-dependent chromatin remodeling factors. Swi2/Snf2-related SWR1 complex in yeast or its corresponding complexes in animals and plants deposits H2A.Z/H2B dimers into nucleosomes in place of H2A/H2B. Oppositely, INO80 complex removes H2A.Z/H2B, incorporating H2A/H2B in return (Morrison and Shen, 2009).

Nucleosome positioning around TSS of genes is important for determining DNA accessibility for RNA polymerase II (Pol II) and other transcription machineries. Therefore, it requires active chromatin remodeling to relocate them to suitable positions for the efficient transcription activities. Genome-wide studies of nucleosome positioning and H2A.Z occupation in yeast and animals have revealed that the regions just upstream of the TSSs are absent from stable nucleosomes. These nucleosome-depleted regions are predominantly flanked by the histone variant H2A.Z throughout the genomes and its formation depends on this proximal H2A.Z (Guillemette et al., 2005; Tirosh and Barkai, 2008; Hu et al., 2013). Because H2A.Z is suggested to inhibit intermolecular association of nucleosomes, it may generate unique chromatin domains poised for transcriptional activation, probably by reducing nucleosome density and increasing DNA accessibility around TSSs (Fan et al., 2002).

In fact, rapid and robust activation of genes, such as oleate-responsive genes, requires SWR1-dependent epigenetic marking with H2A.Z, which is called Htz1 in *Saccharomyces cerevisiae* (Wan et al., 2009). Htz1-containing nucleosomes localizes at promoters of these genes in their repressed states, but they are disassembled upon initial exposure to oleic acid, leading to the loss of Htz1 from the promoters. The nucleosomes reassemble at later stages of gene expression without containing Htz1 protein. Furthermore, in the absence of Htz1, TATA-binding protein (TBP) is not efficiently recruited to oleate-inducible promoters (Table 1). Thus, the presence of Htz1 before induction appears to mark the promoter for sustained gene expression and the recruitment of TBP in yeast.

**Table 1:** **The lists of the chromatin marks and the phenotypes of the mutants written in this review**.

**Chromatin marks**	**Organisms**	**Mutant name**	**Complex name and function**	**Mutant phenotype**
H2A.Z	*Arabidopsis*	*h2a.z*	–	Early flowering, spontaneous cell death, increased pathogen resistance, transcriptional misregulation of responsive genes
		*pie1*	SWR1 complex/H2A.Z incorporation	Early flowering, spontaneous cell death, increased pathogen resistance
		*sef*	SWR1 complex/H2A.Z incorporation	Early flowering, spontaneous cell death, increased pathogen resistance
		*arp6*	SWR1 complex/H2A.Z incorporation	Early flowering, spontaneous cell death, phenocopy of warm grown plants, and phosphate starved plants
	Yeast	*htz1 (h2a.z)*	–	Reduced oleate-response and TBP recruitment
		*ino80*	INO80 complex/Htz1 (H2A.Z) eviction	Genome-wide mislocalization of Htz1, reduced responsiveness of KAR4 gene
	Human	*h2a.z*	–	Decrease in binding of OCT4, MLL and PRC2 to their target genes
H3K4me3	*Arabidopsis*	*atx1*	ATX1 complex/H3K4me3 modification	Attenuation of transcriptional memory of drought stress responsive genes
	Yeast	*set1*	SET1 complex/H3K4me3 modification	Transcriptional memory loss between mother and daughter cells
		*ash2*	SET1 complex/H3K4me3 modification	Transcriptional memory loss between mother and daughter cells
H2Bub	*Arabidopsis*	*hub1*	–/H2Bub modification	Weaker light stimuli response

Mislocalization of H2A.Z also causes altered gene responsiveness. The yeast *ino80* mutant which is deficient in Htz1 removal shows genome-wide mislocalization of H2A.Z, especially with enhanced H2A.Z levels at promoters. This mislocalization leads to the reduced responsiveness to transcriptional changes for cell-cycle dependent gene *KAR4* (Papamichos-Chronakis et al., 2011) (Table 1). These indicate that INO80 dependent H2A.Z eviction at gene promoters is required for efficient gene induction.

The roles of H2A.Z for establishing histone modifications have also been reported. Deficiency in H2A.Z in self-renewing murine embryonic stem cells (ESCs) leads to a decrease in binding of transcription factor OCT4 to its target sites and MLL histone H3K4 methyltransferase complexes to active genes (Hu et al., 2013) (Table 1). H2A.Z knockdown also causes reduction in binding of PRC2 histone H3K27 methyltransferase complex to their target sites to repress the genes. In addition, inhibition of H2A.Z also impairs the signal-induced transcription factor binding, activation of differentiation markers, and the repression of pluripotency genes in differentiating ESCs (Hu et al., 2013). Because, in both cases, H2A.Z defects causes both activation and repression of genes, it is not likely that H2A.Z directly enhances transcription itself nor recruits some specific transcription factors and regulators. Rather, it is suggested that H2A.Z serves as a general facilitator of chromatin accessibility at TSSs for transcriptional modulators including both activating and repressing complexes.

## Functions of H2A.Z in plants

H2A.Z has also important roles in regulating gene expressions in plants. In a forward genetic screen, *ARP6* gene that encodes a subunit of SWR1 complex was identified as an essential factor regulating proper responses to increased temperature in *Arabidopsis* (Kumar and Wigge, 2010) (Table 1). In *arp6* mutant, which is deficient in H2A.Z incorporation, the temperature-responsive genes constitutively respond at normal temperature (22°C), thereby exhibiting the phenotypes of warm grown plants. In wild-type plants, H2A.Z enriches at the first (+1) nucleosome position from TSS of the temperature responsive genes under normal temperature. At warmer temperature (27°C), +1 H2A.Z is decreased and replaced by canonical H2A. Furthermore, the H2A.Z loss is observed both transcriptionally activated and repressed genes after signals of ambient temperature. These data suggest that presence of H2A.Z near TSSs contributes to the responsiveness of genes also in *Arabidopsis*.

Similar phenomena are observed in phosphate starvation response (Smith et al., 2010) and systemic acquired response (March-Díaz et al., 2008) in *Arabidopsis* (Table 1). In *arp6* mutant, phosphate starvation response genes are derepressed without phosphate starving condition, and the plants displays the phenotypes related to phosphate starvation. These genes contain H2A.Z nucleosomes near their TSSs and they are expressed at very low levels under normal conditions in wild-type plants (Smith et al., 2010). Systemic acquired resistance genes are normally expressed depending on plant hormone salicylic acid, but they are constitutively expressed in the *h2a.z* mutant and SWR complex component mutants named *pie1* and *sef*. These plants showed spontaneous cell death and enhanced pathogen resistance. Importantly, in these examples, transcriptional changes in *h2a.z* or *swr1* complex mutant occurred in a same direction as the wild-type plants did when the signals are present. Taken together, these examples suggest that H2A.Z likely serves to keep genes silent in the absence of the inducing signals.

## H2A.Z at gene bodies

Genomic analysis of H2A.Z localization in *Arabidopsis* have revealed that, as other organisms, H2A.Z is primarily enriched at +1 nucleosomes from TSSs. Unlike other organisms, H2A.Z is also found in bodies of genes with low transcription levels (Zilberman et al., 2008). Interestingly, Coleman-Derr et al. found that the deposition of H2A.Z nucleosomes in gene bodies, rather than at TSSs, is correlated higher measures of gene responsiveness (Coleman-Derr and Zilberman, 2012). H2A.Z is enriched within the bodies of genes that respond to the environment or developmental stimuli and *H2A.Z* disruption causes transcriptional misregulation of such genes (Table 1). The authors propose that H2A.Z deposition in gene bodies promotes variability of gene expression.

It is known that upon gene induction, dynamic nucleosome remodeling occurs in stress responsive genes, causing significant changes in nucleosome density and histone modifications in *Arabidopsis* (Chinnusamy and Zhu, 2009; Kim et al., 2010). H2A.Z may be involved at some extent in this alteration of chromatin dynamics around gene bodies of stress responsive genes. Conversely, gene induction can also trigger histone replacement. Another conserved histone variant H3.3 is incorporated into promoters and gene bodies of transcriptionally active genes in *Arabidopsis*, and it is suggested to play a role in histone replacement (Wollmann et al., 2012). Nucleosomes containing both H2A.Z and H3.3 show increased instability compared with canonical H2A/H3 or H2A.Z/H3 in human cells (Jin and Felsenfeld, 2007; Jin et al., 2009). This instability of H3.3/H2A.Z containing nucleosome may facilitate the nucleosome removal in gene bodies, and hence promoting chromatin remodeling and histone modifications to create active chromatin structure appropriate for active transcription. It could also be possible that the instability itself causes sensitivity to the change in intracellular environments such as salt concentration and thereby acts as a sensor for the cellular or extracellular signals.

## DNA methylation prevents H2A.Z incorporation?

Genomewide profiling of H2A.Z and DNA methylation have suggested the global anticorrelation between H2A.Z occupancy and DNA methylation in diverse organisms such as mammals, fish, some insects and plants (Zilberman et al., 2008; Zemach et al., 2010). DNA methylation at first exon of genes, rather than promoter methylation, strictly inhibits transcription initiation in human (Brenet et al., 2011). Consistently, DNA methylation is nearly absent around TSSs of genes in many organisms especially when the genes are active whereas TSS DNA methylation does not show remarkable decrease for the genes with low expression (Lister et al., 2008; Feng et al., 2010; Zemach et al., 2010). Therefore, antagonistic relationship between DNA methylation and H2A.Z at TSSs may decide whether the gene is silenced or kept poised for activation. Recent genomic bisulfite sequencing analyses have shown that DNA methylation is found not only in transposons or silent gene promoters but also within transcribed genes in many eukaryotes (Feng et al., 2010; Zemach et al., 2010). Interestingly, this gene body methylation also globally anticorrelates with H2A.Z occupation in gene bodies in *Arabidopsis* (Zilberman et al., 2008). Gene body methylation shows clear anticorrelation with gene responsiveness and it is often found in housekeeping genes (Aceituno et al., 2008). It is not clear whether this anticorrelation is the direct effect of gene body methylation or indirect effect probably through preventing of H2A.Z incorporation. Because of its clear and global anticorrelation, it has been suggested that DNA methylation and H2A.Z exclude each other (Zilberman et al., 2008). In fact, the global demethylation in *Arabidopsis met1* CpG methyltransferase mutant causes increase in H2A.Z occupancies within the wild-type methylated regions (Zilberman et al., 2008). However, disruption of *H2A.Z* coding genes or global elimination of H2A.Z in *Arabidopsis* mutants does not lead to the increased levels of DNA methylation in genes (Coleman-Derr and Zilberman, 2012). Therefore, the authors propose that the anticorrelation between H2A.Z and DNA methylation is caused by exclusion of H2A.Z from methylated DNA. It will be interesting whether loss of gene body methylation causes the incorporation of H2A.Z within those genes and, if that is the case, results in the increase in transcription instability.

## Transcriptional memory

Another strategy to regulate gene responsiveness is to memorize the recent activity of transcription status. The memory can be stored by specific epigenetic information. One such information is histone H3K4 methylation. H3K4me3 is a prominent histone mark that is associated with gene promoters in many eukaryotes (Schneider et al., 2004; Vermeulen and Timmers, 2010). Promoter H3K4me3 is directly and very selectively recognized by TAF3, a subunit of the basal transcription initiation factor TFIID, via its plant homeodomain (PHD) finger domain in animals. H3K4me3 thus plays a role in Pol II recruitment to active gene promoters (Vermeulen et al., 2007; Lauberth et al., 2013). Correspondingly, computational analysis of the relationship between core promoter locations and histone modifications showed that the H3K4 methylation marks are strongly predictive for promoter location in human (Chen et al., 2011). In addition, mutations in TAF3 PHD finger domain or TATA-box in human cells incapacitated the assembly of transcription preinitiation complex and p53-dependent rapid induction (Lauberth et al., 2013).

Although H3K4me3 is clearly correlated with gene activity, this modification seems to be established downstream of transcription activity. Thus, it has been proposed that H3K4 methylation could serve not only for a recruiter of Pol II but also for epigenetic memory of recent transcriptional activity. This memory of transcription seems to be transmittable through generations in yeast; transcription activities are very similar between mother and daughter cells (Muramoto et al., 2010). The components of histone H3K4 methyltransferase complex Set1 and Ash2 are required for this transcriptional memory as the mutants in these genes fails to maintain the transcription frequency (Table 1). It is also observed by the amino acid substitution from H3K4 to alanine, suggesting that the transcriptional memory between generations may be mediated directly by H3K4 methylation.

## Transcriptional memory in plants

H3K4me3 is predominantly found around promoter and 5′ regions of genes in plants, as is the case for yeast and mammals (Zhang et al., 2009). H3K4me3 in gene bodies has been suggested to play a role in transcriptional memory in *Arabidopsis* (Alvarez-Venegas et al., 2007; Jaskiewicz et al., 2011). H3K4me3 is increased within gene bodies during gene induction of some drought responsive genes. The timing of H3K4me3 enrichment followed after Pol II enrichment, which is consistent with the dispensability of H3K4me3 for the initiation of transcription (Kim et al., 2008). Interestingly, recent two papers presented the evidences that the enriched H3K4me3 in gene bodies decreased after stress recovery but remained at some levels, suggesting that H3K4me3 may contribute for the transcriptional memory in *Arabidopsis* (Ding et al., 2012; Kim et al., 2012). Consistently, some drought responsive genes such as *RD29B* and *RAB18* showed enhanced induction after experience of the first induction (Ding et al., 2012). In addition, this transcriptional memory was attenuated in the *Arabidopsis* H3K4 methyltransferase mutant *atx1* (Table 1). In contrast, other active chromatin modifications such as acetylation of histone H3 and H4 were coordinated with gene expression levels; rapidly increased upon induction and quickly decreased after the genes being repressed and become at comparable levels as before induction (Ding et al., 2012; Kim et al., 2012). These results suggest the role of gene body H3K4me3 in establishing transcriptional memory.

Likewise, a role of transcriptional memory was also suggested for Histone H2B monoubiquitination (H2Bub), a mark linked to transcription elongation. H2Bub has been suggested to act upstream of H3K4me3 in yeast (Sun and Allis, 2002) and it facilitates rapid modulation of gene expression during photomorphogenesis in *Arabidopsis* (Bourbousse et al., 2012). Genome-wide H2Bub distribution together with transcription profiling in *Arabidopsis* revealed that H2Bub levels increases over gene bodies in concert with gene up-regulation, whereas H2Bub generally remains stable during gene down-regulation. Lacking H2Bub in *hub1* mutant results in weaker responses of genes to light stimuli (Table 1). H2Bub affects responsiveness of genes whose expression is rapidly and transiently altered by light signals such as circadian related genes. It remains unexplored whether this possible transcriptional memory with H2Bub is directly connected to H3K4me3 in gene bodies.

In eukaryotes, because of the spatial separation of transcription and translation by nuclear envelope, the association of active genes to nuclear envelope facilitates efficient transcription and subsequent mRNA transport, which is regulated by nuclear pore complexes. In yeast and human cells, some inducible genes physically interact with nucleoporin Nup100 (yeast) or Nup98 (human) upon activation and also for several generations after subsequent repression (Gialitakis et al., 2010; Arib and Akhtar, 2011; Light et al., 2013). During the repression period, Pol II and H3K4 methylation remain in the promoter, but they are lost in the absence of nucleoporins, resulting in reduced gene responsiveness. Thus, it is suggested that the binding of Nup100/Nup98 to recently activated promoters plays a conserved role in epigenetic transcriptional memory. It would be interesting to learn whether plants also regulate nuclear localization of inducible genes with similar mechanisms and whether the H3K4me3 in gene bodies is involved in this process.

## Conclusion

Gene responsiveness affects numerous biological processes. To achieve proper gene responsiveness, it is essential to regulate particular chromatins around genes. Recent epigenetic studies in *Arabidopsis* have proposed that some specific chromatins in gene bodies, rather than around TSSs, are required to set up proper gene responsiveness in plants. The occupancy of histone variant H2A.Z in gene bodies is correlated with gene responsiveness and is required for keeping genes poised to respond. In addition, H3K4me3 in gene bodies is suggested to have a role in transcriptional memory. Although the molecular mechanisms that underlie these observations remain undefined, these findings raise many interesting questions. Are these two epigenetic marks co-localized and/or interact? How long do these epigenetic memories persist and which factors are involved in this memory maintenance or erasure? In addition, it is also of great interest whether these epigenetic dynamics in gene bodies observed in *Arabidopsis* is applicable to genes in other organisms.

### Conflict of interest statement

The authors declare that the research was conducted in the absence of any commercial or financial relationships that could be construed as a potential conflict of interest.

## Abbreviations

ARP6: Actin-Related Protein 6
ASH2: Absent, small, or homeotic discs 2
INO80: Inositol requiring 80
HTZ1: Histone Two A Z1
MLL: Mixed-Lineage Leukemia or Myeloid-Lymphoid Leukemia;OCT4, Octamer-binding Transcription Factor 4
PIE1: Photoperiod-Independent Early Flowering 1
PRC2: Polycomb Repressive Complex 2
SEF: Serrated Leaves and Early Flowering
SET1: SET domain containing 1
SWR1: Sick With Rat8 ts 1
TAF3: TATA binding protein associated factor 3.

## References

[B1] Aceituno F. F. Moseyko N. Rhee S. Y. Gutiérrez R. A. (2008). The rules of gene expression in plants: organ identity and gene body methylation are key factors for regulation of gene expression in *Arabidopsis thaliana*. BMC Genomics 9:438 10.1186/1471-2164-9-438 18811951 PMC2566314

[B2] Alvarez-Venegas R. Abdallat A. A. Guo M. Alfano J. R. Avramova Z. (2007). Epigenetic control of a transcription factor at the cross section of two antagonistic pathways. Epigenetics 2, 106–113 10.4161/epi.2.2.4404 17965588

[B3] Arib G. Akhtar A. (2011). Multiple facets of nuclear periphery in gene expression control. Curr. Opin. Cell Biol. 23, 346–353 10.1016/j.ceb.2010.12.005 21242077

[B4] Bourbousse C. Ahmed I. Roudier F. Zabulon G. Blondet E. Balzergue S. (2012). Histone H2B monoubiquitination facilitates the rapid modulation of gene expression during Arabidopsis photomorphogenesis. PLoS Genet. 8:e1002825 10.1371/journal.pgen.1002825 22829781 PMC3400566

[B5] Brenet F. Moh M. Funk P. Feierstein E. Viale A. J. Socci N. D. (2011). DNA methylation of the first exon is tightly linked to transcriptional silencing. PLoS ONE 6:e14524 10.1371/journal.pone.0014524 21267076 PMC3022582

[B6] Chen Y. Jørgensen M. Kolde R. Zhao X. Parker B. Valen E. (2011). Prediction of RNA Polymerase II recruitment, elongation and stalling from histone modification data. BMC Genomics 12:544 10.1186/1471-2164-12-544 22047616 PMC3228824

[B7] Chinnusamy V. Zhu J. K. (2009). Epigenetic regulation of stress responses in plants. Curr. Opin. Plant Biol. 12, 133–139 10.1016/j.pbi.2008.12.006 19179104 PMC3139470

[B8] Coleman-Derr D. Zilberman D. (2012). Deposition of histone variant H2A.Z within gene bodies regulates responsive genes. PLoS Genet. 8:e1002988 10.1371/journal.pgen.1002988 23071449 PMC3469445

[B9] Deal R. B. Henikoff S. (2011). Histone variants and modifications in plant gene regulation. Curr. Opin. Plant Biol. 14, 116–122 10.1016/j.pbi.2010.11.005 21159547 PMC3093162

[B10] Ding Y. Fromm M. Avramova Z. (2012). Multiple exposures to drought “train” transcriptional responses in *Arabidopsis*. Nat. Commun. 3, 740 10.1038/ncomms1732 22415831

[B11] Fan J. Y. Gordon F. Luger K. Hansen J. C. Tremethick D. J. (2002). The essential histone variant H2A.Z regulates the equilibrium between different chromatin conformational states. Nat. Struct. Biol. 9, 172–176 10.1038/nsb767 11850638

[B12] Feng S. Cokus S. J. Zhang X. Chen P. Y. Bostick M. Goll M. G. (2010). Conservation and divergence of methylation patterning in plants and animals. Proc. Natl. Acad. Sci. U.S.A. 107, 8689–8694 10.1073/pnas.1002720107 20395551 PMC2889301

[B13] Gialitakis M. Arampatzi P. Makatounakis T. Papamatheakis J. (2010). Gamma interferon-dependent transcriptional memory via relocalization of a gene locus to PML nuclear bodies. Mol. Cell Biol. 30, 2046–2056 10.1128/MCB.00906-09 20123968 PMC2849471

[B14] Guillemette B. Bataille A. R. Gévry N. Adam M. Blanchette M. Robert F. (2005). Variant histone H2A.Z is globally localized to the promoters of inactive yeast genes and regulates nucleosome positioning. PLoS Biol. 3:e384 10.1371/journal.pbio.0030384 16248679 PMC1275524

[B15] Hu G. Cui K. Northrup D. Liu C. Wang C. Tang Q. (2013). H2A.Z facilitates access of active and repressive complexes to chromatin in embryonic stem cell self-renewal and differentiation. Cell Stem Cell 12, 180–192 10.1016/j.stem.2012.11.003 23260488 PMC3570599

[B16] Jaskiewicz M. Conrath U. Peterhänsel C. (2011). Chromatin modification acts as a memory for systemic acquired resistance in the plant stress response. EMBO Rep. 12, 50–55 10.1038/embor.2010.186 21132017 PMC3024125

[B17] Jin C. Felsenfeld G. (2007). Nucleosome stability mediated by histone variants H3.3 and H2A.Z. Genes Dev. 21, 1519–1529 10.1101/gad.1547707 17575053 PMC1891429

[B18] Jin C. Zang C. Wei G. Cui K. Peng W. Zhao K. (2009). H3.3/H2A.Z double variant-containing nucleosomes mark “nucleosome-free regions” of active promoters and other regulatory regions. Nat. Genet. 41, 941–945 10.1038/ng.409 19633671 PMC3125718

[B19] Kim J. M. To T. K. Ishida J. Matsui A. Kimura H. Seki M. (2012). Transition of chromatin status during the process of recovery from drought stress in *Arabidopsis thaliana*. Plant Cell Physiol. 53, 847–856 10.1093/pcp/pcs053 22505693

[B20] Kim J. M. To T. K. Ishida J. Morosawa T. Kawashima M. Matsui A. (2008). Alterations of lysine modifications on the histone H3 N-tail under drought stress conditions in *Arabidopsis thaliana*. Plant Cell Physiol. 49, 1580–1588 10.1093/pcp/pcn133 18779215

[B21] Kim J. M. To T. K. Nishioka T. Seki M. (2010). Chromatin regulation functions in plant abiotic stress responses. Plant Cell Environ. 33, 604–611 10.1111/j.1365-3040.2009.02076.x 19930132

[B22] Kumar S. V. Wigge P. A. (2010). H2A.Z-containing nucleosomes mediate the thermosensory response in *Arabidopsis*. Cell 140, 136–147 10.1016/j.cell.2009.11.006 20079334

[B23] Lauberth S. M. Nakayama T. Wu X. Ferris A. L. Tang Z. Hughes S. H. (2013). H3K4me3 interactions with TAF3 regulate preinitiation complex assembly and selective gene activation. Cell 152, 1021–1036 10.1016/j.cell.2013.01.052 23452851 PMC3588593

[B24] Light W. H. Freaney J. Sood V. Thompson A. D'Urso A. Horvath C. M. (2013). A conserved role for human Nup98 in altering chromatin structure and promoting epigenetic transcriptional memory. PLoS Biol. 11:e1001524 10.1371/journal.pbio.1001524 23555195 PMC3608542

[B25] Lister R. O'Malley R. C. Tonti-Filippini J. Gregory B. D. Berry C. C. Millar A. H. (2008). Highly integrated single-base resolution maps of the epigenome in *Arabidopsis*. Cell 133, 523–536 10.1016/j.cell.2008.03.029 18423832 PMC2723732

[B26] March-Díaz R. García-Domínguez M. Lozano-Juste J. León J. Florencio F. J. Reyes J. C. (2008). Histone H2A.Z and homologues of components of the SWR1 complex are required to control immunity in Arabidopsis. Plant J. 53, 475–487 10.1111/j.1365-313X.2007.03361.x 17988222

[B27] Morrison A. J. Shen X. (2009). Chromatin remodelling beyond transcription: the INO80 and SWR1 complexes. Nat. Rev. Mol. Cell Biol. 10, 373–384 10.1038/nrm2693 19424290 PMC6103619

[B28] Muramoto T. Müller I. Thomas G. Melvin A. Chubb J. R. (2010). Methylation of H3K4 Is required for inheritance of active transcriptional states. Curr. Biol. 20, 397–406 10.1016/j.cub.2010.01.017 20188556

[B29] Papamichos-Chronakis M. Watanabe S. Rando O. J. Peterson C. L. (2011). Global regulation of H2A.Z localization by the INO80 chromatin-remodeling enzyme is essential for genome integrity. Cell 144, 200–213 10.1016/j.cell.2010.12.021 21241891 PMC3035940

[B30] Raisner R. M. Madhani H. D. (2006). Patterning chromatin: form and function for H2A.Z variant nucleosomes. Curr. Opin. Genet. Dev. 16, 119–124 10.1016/j.gde.2006.02.005 16503125

[B31] Schneider R. Bannister A. J. Myers F. A. Thorne A. W. Crane-Robinson C. Kouzarides T. (2004). Histone H3 lysine 4 methylation patterns in higher eukaryotic genes. Nat. Cell Biol. 6, 73–77 10.1038/ncb1076 14661024

[B33] Smith A. P. Jain A. Deal R. B. Nagarajan V. K. Poling M. D. Raghothama K. G. (2010). Histone H2A.Z regulates the expression of several classes of phosphate starvation response genes but not as a transcriptional activator. Plant Physiol. 152, 217–225 10.1104/pp.109.145532 19897606 PMC2799358

[B34] Sun Z. W. Allis C. D. (2002). Ubiquitination of histone H2B regulates H3 methylation and gene silencing in yeast. Nature 418, 104–108 10.1038/nature00883 12077605

[B35] Suto R. K. Clarkson M. J. Tremethick D. J. Luger K. (2000). Crystal structure of a nucleosome core particle containing the variant histone H2A.Z. Nat. Struct. Biol. 7, 1121–1124 10.1038/81971 11101893

[B36] Tirosh I. Barkai N. (2008). Two strategies for gene regulation by promoter nucleosomes. Genome Res. 18, 1084–1091 10.1101/gr.076059.108 18448704 PMC2493397

[B37] Vermeulen M. Mulder K. W. Denissov S. Pijnappel W. W. van Schaik F. M. Varier R. A. (2007). Selective anchoring of TFIID to nucleosomes by trimethylation of histone H3 lysine 4. Cell 131, 58–69 10.1016/j.cell.2007.08.016 17884155

[B38] Vermeulen M. Timmers H. T. (2010). Grasping trimethylation of histone H3 at lysine 4. Epigenomics 2, 395–406 10.2217/epi.10.11 22121900

[B39] Wan Y. Saleem R. A. Ratushny A. V. Roda O. Smith J. J. Lin C. H. (2009). Role of the histone variant H2A.Z/Htz1p in TBP recruitment, chromatin dynamics, and regulated expression of oleate-responsive genes. Mol. Cell Biol. 29, 2346–2358 10.1128/MCB.01233-08 19273605 PMC2668375

[B40] Wollmann H. Holec S. Alden K. Clarke N. D. Jacques P.É. Berger F. (2012). Dynamic deposition of histone variant H3.3 accompanies developmental remodeling of the *Arabidopsis* transcriptome. PLoS Genet. 8:e1002658 10.1371/journal.pgen.1002658 22570629 PMC3342937

[B41] Zemach A. McDaniel I. E. Silva P. Zilberman D. (2010). Genome-wide evolutionary analysis of eukaryotic DNA methylation. Science 328, 916–919 10.1126/science.1186366 20395474

[B42] Zhang X. Bernatavichute Y. V. Cokus S. Pellegrini M. Jacobsen S. E. (2009). Genome-wide analysis of mono-, di- and trimethylation of histone H3 lysine 4 in *Arabidopsis thaliana*. Genome Biol. 10:R62 10.1186/gb-2009-10-6-r62 19508735 PMC2718496

[B43] Zilberman D. Coleman-Derr D. Ballinger T. Henikoff S. (2008). Histone H2A.Z and DNA methylation are mutually antagonistic chromatin marks. Nature 456, 125–129 10.1038/nature07324 18815594 PMC2877514

